# *Reviving the forgotten:* breathing life into urban wastelands through skateboarding and decolonial placemaking in Nairobi, Kenya

**DOI:** 10.3389/fspor.2025.1637588

**Published:** 2025-11-07

**Authors:** Athanasios Tsipis, Sepandarmaz Mashreghi

**Affiliations:** 1 Malmö University, Malmö, Sweden; 2 Norwegian University of Science and Technology, Trondheim, Norway

**Keywords:** affective ecologies, relational world-making, wastelands, youth cultures, community care, urban commons

## Abstract

Skateboarding in Nairobi, Kenya, offers young people ways of asserting subjectivity, reimagining the city through movement, care, and shared presence. Based on semi-structured interviews, field observations, and sensory ethnography, this study traces how skateboarders transform overlooked sites—plazas, rooftops, and improvised parks—into spaces of community building, ecological care, and affective belonging. Grounded in decolonial thought, Indigenous teachings, and feminist ethics of care, the analysis frames skateboarding as a collective practice through which personhood is shaped, care for community and environment is sustained, and otherwise ways of being in the city are made possible. The findings show how Nairobi's skateboarders negotiate colonial and patriarchal histories and structures, generate affective ecologies of belonging through sound and movement, and practice feminist placemaking through accountability and solidarity. Clean-ups, DIY ramp-building, and the reclaiming of wastelands illustrate how skaters convert abandonment into commons and environmental responsibility. Such practices are not without tension, as skateboarders navigate precarity, layered marginalization, policing, stigmatization, the absence of formal facilities, complicity and conflicts within their own collectives. Ultimately this paper demonstrates that Nairobi's skateboarding practices are not only leisure, but also affective and relational world-making that point toward alternative decolonial urban futures.

## 1 Introduction

On a humid evening at The Mall's rooftop in Nairobi, two teenage boys in scuffed sneakers took turns attempting a difficult rail slide. A core of a few dozen skaters and interested spectators cheered in Sheng and Kiswahili. When the board leaped out under one of them, he tumbled, laughed, and was instantly picked up by some friends. One heard laughter, claps, and utterances such as like “utapata next time” (you'll get it next time) filled the air. They are examples of our field observations, in which skateboarding transfigures the unused rooftop locations into interactive multi-purpose areas of assistance and improvisation.

Nairobi is a rapidly growing city where roughly three-quarters of the population is below 35 years old; many youths gain their income in non-formal jobs (1, 2). Local idioms like *tarmacking* and *hustling* describe the search for employment and survival in a context of infrastructural neglect and economic precarity. Skaters navigate this unpredictability by carving out spaces for leisure and community on the margins. They often describe skateboarding as a way of “moving ahead”—both literally and figuratively—through the city's uneven streets.

This paper brings the voices and experiences of the skaters in Nairobi to the fore. We inductively build our analysis from ethnographic scenes to explore how skateboarding becomes a practice of agency, belonging, and care, for both their community and their habitat, among Nairobi's youth. We ask: How do skaters reconfigure grey spaces into sites of possibility? How do their embodied and affective practices contribute to decolonial placemaking and youth agency in Kenya? By centering on lived experience, we situate skateboarding within the broader debates on the city's aspirations, precariousness, and social transformation in Nairobi.

Skateboarding has evolved from its 1950s Californian roots as a surfers' pastime into a global urban phenomenon that embodies creativity, resistance, and spatial subversion. While Western scholarship has extensively documented its role in youth identity formation and urban placemaking, little attention has been given to its socio-cultural and environmental implications in post-colonial African cities. This paper addresses that gap through a decolonial engagement with the concepts of care and community, contextualizing Nairobi's skateboarding culture. It illustrates how skateboarders reclaim abandoned urban spaces—such as derelict parks and rooftops—as acts of radical (self) care, spatial sovereignty, and environmental consciousness. It also challenges masculinist narratives in skateboarding literature by centering the experiences of a gender-diverse sample of Nairobi skaters. Ultimately, this study explores how skateboarding in Nairobi facilitates decolonial placemaking, youth agency, alternative belongings, and grassroots climate action, thereby countering Eurocentric urban paradigms. Central to this investigation is the affective experience of skateboarding—how it generates feelings of care, freedom, tension, (non)belonging, and joy—and how these emotions shape young people's relationships to urban space and to one another.

Recent Kenyan ethnographies shed light on local survival strategies that shape the livelihoods and aspirations of young people in Kenya. Rahier's study of Nakuru describes *tarmacking* as a tedious yet vital practice through which young people navigate infrastructural neglect and economic uncertainty via ceaseless movement and improvisation (3). Thieme's work on *hustling* in Nairobi explores how youth cultivate relational networks and tactical improvisation to survive the *mtaa* (street) economy, foregrounding the importance of social ties and adaptive creativity in precarious environments (4). Chułek (5) shows that slum dwellers, engaged in waste collection and recycling, organize themselves into informal *orodha* (lists) and rotational systems that distribute labor and resources, while producing networks of care and solidarity in Nairobi's slums. Van Stapele (6) analyzes how youth in informal neighborhoods negotiate aspiration, danger and gendered respectability, revealing how care and belonging are continuously negotiated within spaces marked by insecurity. These Kenyan studies ground our analysis by tracing the socio-cultural rhythms of *tarmacking*, *hustling*, and *orodha* that shape Nairobi's urban youth experience, while illuminating how skateboarders draw on these energies to reconfigure grey spaces and imagine alternative futures.

Nairobi's contemporary landscapes cannot be understood without acknowledging the city's colonial foundations. British planners imagined Nairobi as a *white city*, diverting sanitation, infrastructure, and public space toward European settlers while pushing African and Indian residents into overcrowded, unsanitary settlements (7, 8). Even today, colonial-era master plans, designed by white South African planners in 1948, continue to structure the city along race and class lines, promoting spacious European-style suburbs and restricting Indigenous communities to the margins (9). This segregation is not just historical memory, but part of Nairobi's lived reality; it shapes how youth from informal settlements and middle-class estates encounter one another and navigate urban space. Understanding this layered coloniality is essential for contextualizing the practices described in this paper.

Recent scholarship demonstrates that skateboarding reclaims and transforms *grey spaces*—underutilized and polluted urban areas—through embodied sensory practice and collective creativity (10). O'Connor and colleagues (10) conceptualize grey spaces as sites of environmental and political ambiguity, where leisure can simultaneously embody sustainability and pollution. In Nairobi, grey spaces include concrete plazas, rooftop carparks, underpasses, and industrial wastelands that lack greenery and are often associated with insecurity and neglect. These sites pose threats to health and community well-being through air pollution, heat stress, and a lack of public amenities. However, as Lee (11) poignantly notes, “*wastelands are named by the ones responsible for their devastation,”* implying that such spaces have value and meaning to those who inhabit them. Skateboarding brings these areas to life and gives participants the ability to re-imagine banal urban architecture—stairs, curbs, and plazas—as playable surfaces that invite attention and care as part of a living urban fabric. This perspective underscores that greyness in Nairobi is not simply aesthetic; it is socio-cultural, reflecting colonial histories of segregation, contemporary dispossession, and youth efforts to reclaim community through movement and care.

For example, Zittel's (12) sensory ethnography shows how embodied experiences—such as the rhythmic sound of wheels on concrete—foster interclass connections and collective spatial practices. These engagements reshape both bodies and environments, with skaters using chalk lines and *skitch* marks to signal shared ownership of neglected sites. Media reports on African skate scenes (13) reinforce these themes, noting that communities across the continent generate networks of mutual support, technical innovation, and resilience even as the decolonial dimensions of their practices remain under-theorized.

Other scholars underscore the political significance of DIY skate spots–as opposed to formal skateparks and structures, which resist capitalist and colonial logics through self-directed, creative spatial use. Hölsgens and Glenney (14) critique the Western focus on identity and aesthetics, calling for greater attention to how skateboarding repurposes colonial infrastructures and contributes to grassroots climate and gender justice. Female skaters, for instance, form collectives to challenge patriarchal norms and claim safer urban space. Romero & Miles (15) frame communal skate sessions as decolonial rituals that draw on Indigenous epistemologies of place, using sensory attunement and vernacular naming to reclaim and re-narrate colonial geographies.

Scholars worldwide recognize that skateboarding can facilitate alternative forms of belonging, resourceful adaptations, and urban revitalization by providing spaces for those who are otherwise marginalized (16, 17).

### 1.1 Skateboarding as decolonizing praxis

Recent scholarship on skateboarding and decolonization emphasizes that Indigenous skaters and communities of color co-create the DIY ethos, reclaiming sovereignty over land and challenging assumptions of unmarked whiteness (16, 17). These perspectives inform our analysis and position Nairobi's skateboarding practices within a broader global movement toward decolonization and Indigenization.

For scholars of skate pedagogy, the ethics of care is central. The edited volume *Skate/worlds: New Pedagogies for Skateboarding* frames skateboarding as a prefigurative learning practice, exploring care work, motherhood, anticolonial pedagogy, and peer-based DIY support. These practices speak directly to the ethical and environmental concerns shaping contemporary societies (18). Learning processes are always situated, and skateboarding communities evolve differently across social and geographic contexts, shaped by intersectional dynamics and critical pedagogies to foreground how gender, race, and class influence access and experience within skateboarding cultures (18). In addition, Geckle (16) highlights skateboarding's decolonizing potential, showing how skateboarding re-imagines urban space, diversifies Indigenous arts and practices, and fosters community-building grounded in visions of Indigenous futures. Kamper and Williams (17) emphasize how Black and Indigenous skaters bring place-based practices into the DIY ethos, challenging its framing as culturally unmarked. Glenney and DuBois (19) connect skateparks to environmental justice, identifying them as sites for contesting pollution and advancing decolonial ecological agendas.

Together, these studies demonstrate that care, pedagogy, and decolonial praxis are deeply entangled in contemporary skateboarding. This work informs our approach to the Nairobi scene, particularly in how we understand the intersections of space, learning, and community. Our work, therefore, explores how Nairobi's skateboarders reclaim the city's grey spaces through practices of care that embody decoloniality—transforming neglected infrastructure into spaces of relation, imagination, and affective belonging.

## 2 Materials and methods

### 2.1 Theoretical approach: decoloniality, care, and the possibility of the *otherwise*

Decoloniality, as articulated by Mignolo, Walsh, and Maldonado-Torres (20–22), frames coloniality as an ongoing matrix of power that orders knowledge, being, and social relations. Decoloniality, hence, is not only a critique of colonial domination but an embodied praxis of living and relating otherwise. Rather than seeking a return to a pre-colonial past, decolonial praxis opens space for the *otherwise*—relational, situated ways of inhabiting the world that unsettle colonial hierarchies through daily practices of refusal, care, and (re)creation (23).

Care, in this context, is not a retreat from politics but a generative force for imagining and sustaining alternative futures. Achille Mbembe argues that decolonization aims not merely at justice but at restoring the will to life and community, enabling new ways of being in the world (24). Erica Violet Lee's extends this perspective by framing the *care of wastelands* as an engagement with polluted and neglected sites, recognizing them as places saturated with story, trauma, and potential; to care for them is to resist abandonment and to engage in relational placemaking that affirms life in the aftermath of dispossession (11). bell hooks similarly defines care as a political ethic of love and connection—foundational to collective survival, and a refusal of alienation and individualism (25). Together, these thinkers position care as a decolonial praxis that grounds the (re)creation of relational, life-affirming futures.

In concert, these frameworks foreground care as an everyday, embodied ethic through which communities tend to themselves, to each other, and to the land. These perspectives inform our study, offering conceptual tools for understanding how care, refusal, and relationality unfold in urban space. In Nairobi's skate scene, the *otherwise* takes shape when youth transform overlooked urban wastelands into sites of relational care, creativity, and joy. These practices enact a decolonial ethic of being together in difference, and of remaking space through collective presence, attention, and possibility.

### 2.2 Methodology

This article draws on data collected as part of a Master's thesis project, completed in 2024, which explored how informal skateboarding in Nairobi shapes urban space, ecological engagement, and social relations (26). The study was conducted in accordance with the protocols of the Swedish Ethical Review Authority; informed consent was obtained from all participants, and anonymization procedures were implemented to protect their identities.

The qualitative approach of sensory and embodied ethnography was employed to capture the lived experiences of skateboarders as they navigate, transform, and claim space through movement and care. Inspired by Zittel's (12) sensory ethnography of Nairobi skateboarders, our study builds on the idea that rhythmic movement, sound, and tactile engagement with urban surfaces foster interpersonal connection and collective claims to space. Rather than functioning as a distinct theoretical lens, sensory anthropology complements decolonial and relational frameworks by directing attention to how sensory practices generate meaning, memory, and attachment in space.

Zittel (12) highlights how the sound of wheels on concrete, the texture of asphalt, and the visual elements of the urban landscape become part of a shared sensory language that cuts across class divisions. In our research, participant observation and audio recordings traced how *skatesound* functioned as an auditory marker of spatial belonging. These sensory interactions revealed how skateboarders map feelings of care, memory, and relational presence onto the city's overlooked or marginal spaces. This approach is further supported by Hölsgens and Glenney's (27) work on skateboarding and sensory anthropology, which argues that skaters acquire somatic knowledge and socio-emotional resilience through the vibratory and sonic experiences of city streets. Sideways movement cultivates a distinct sensorium attuned to urban architecture, while repeated falls and injuries form part of a pedagogy of care—one that encourages slower, less harmful, and more community-oriented styles of skating. Together, these methodological tools enable a nuanced understanding of how Nairobi's skateboarders inhabit and remake space, not only through visible actions but also through the felt, heard, and lived dimensions of everyday urban movement.

#### 2.2.1 Study context

Nairobi, with a population of over 4.8 million and an annual growth rate of 3.94% (28), is characterized by rapid urbanization and deep spatial inequalities. Over 60% of the city's residents live in informal settlements, such as Kibera and Korogocho, where overcrowding and limited public infrastructure restrict recreational opportunities (28, 29). In these conditions, skateboarding operates as a practice of collective appropriation that reworks underused urban surfaces into sites of creativity and community (30–32).

This study focuses on two contrasting environments that illustrate different spatial strategies. Shangilia Skatepark, a formal facility established by Skate-Aid within the Shangilia slum orphanage, provides mentorship, safety, and community support (33). Opened in 2013, it is the largest purpose-built skatepark in East Africa (34), aligning with UN-Habitat's New Urban Agenda and Nairobi's 2025 urban development goals (35). In contrast, improvised urban sites—such as Uhuru Park platforms and The Mall rooftop—are tactically repurposed by skaters through spontaneous interventions that subvert normative planning and transform grey infrastructure into spaces of belonging.

#### 2.2.2 Participants

Participants were 10 active skateboarders (5 male, 5 female), ages 18–32, recruited through purposive and snowball sampling from grassroots skate collectives, including GirlSkate Nairobi, She Skates Kenya, and the Skateboarding Society of Kenya. This gender-balanced group reflects the city's youthful demographics and aligns with intersectional urban research methodologies (36). Participants varied in socio-economic status, with 40% working in the informal sector, reflecting patterns like the 62% of Korogocho residents engaged in gig economies (28). Thirty percent were active in environmental and gender justice work, including collaborations with the Awoche Foundation (37).

#### 2.2.3 Handling of data

Data collection was conducted by the first author and included over 80 h of participant observation and interviews at both formal and improvised skateboarding sites, such as Shangilia Skatepark and street spots like The Mall rooftop. Fieldwork focused on how skaters negotiate, repurpose, and care for urban environments. Interview prompts explored themes such as gender dynamics, ecological stewardship, and emotional relationships to community and to place. Shared reflections with participants (member-checking) ensured ethical accountability and representational accuracy. The semi-structured interviews were audio-recorded with consent and transcribed verbatim. Analysis followed a reflexive thematic approach (38), with initial inductive coding led by the first author. The empirical material was later reframed and analyzed collaboratively by both authors through decolonial theoretical lenses. This dialogical process enabled a layered interpretation grounded in practice, while remaining attentive to the broader political and relational dimensions of urban life.

### 2.3 Researcher positionality and rolling ethnography

The first author has been an active skateboarder for over two decades and entered the field as a partial insider. While this long-term experience provided technical familiarity and cultural insight, he limited his skating during fieldwork to occasional sessions for rapport-building and instead prioritized observation, note-taking, and semi-structured interviews. This nuanced engagement draws on *rolling ethnography*, a methodological sensibility wherein skateboarding itself becomes a legitimate research tool alongside walking, talking, and listening (39). By skating just enough to understand the flow of lines and the feel of rough concrete while remaining largely on the periphery, the first author balanced insider knowledge with analytic distance. The second author contributed as supervisor and co-author, offering a distanced perspective grounded in decolonial research practices. While not a skateboarder, her role in shaping the theoretical framework and guiding the analytic process helped situate the study within broader questions of care, space, and community. Our positionality, therefore, combined participatory insight with careful observation to capture the embodied and relational textures of Nairobi's skate scenes.

## 3 Findings and discussion

### 3.1 Self-care and embodied agency

Skateboarding in Nairobi fosters not only individual agency but also collective urban transformation. Skaters' bodily movements and affective attachments enacted an alternative form of care that converged in everyday practices of enacting agency and self-making. Participants described skateboarding as a powerful source of courage, agency, and self-care in their everyday lives. Emily reflected, “*When I try new tricks… I'm like, I can do this. If I can do this on the board, I can do other things in life.”* Similarly, Ben noted, “*Every fall is a lesson, and every trick landed is a victory,”* emphasizing the iterative process of failure and success as a personal philosophy. James, a veteran skater of 15 years, shared, “*I used to be indoors [shy]… Skating taught me commitment, to be fearless.”* He described how repeatedly falling and getting back up “trained” him to persist through life's difficulties. These narratives reveal how the embodied act of skateboarding becomes a vehicle for enacting one's agency in life, beyond skateboarding. Mashreghi (40) explores similar themes, demonstrating how the mastery of cycling becomes a site for constructing personhood, cultivating agency, and affirming self-worth among migrant youths. At the same time, skateboarding here functions as a mode of self-care and collective healing. Ben described it as “*my escape when things weren't going right, that sense of belonging, that sense of, maybe, I can overcome challenges.”* James echoed this: “*Shredding just takes your mind off stuff, and you don't think about all the stressors that are going on.”* These accounts underscore the affective role of skateboarding as a site of what Lorde (41) famously framed as an act of radical self-care—a deliberate, sustaining practice of self-care in contexts that routinely devalue marginalized lives. Furthermore, Nairobi skateboarders view their decks as canvases for self-portraiture, merging athleticism with personal narrative and creative expression. Charlotte reflected, “*Every trick landed is a reminder of what I can achieve if I put my mind to it,”* while Henry likened trick execution to his profession as a DJ: “*Doing a trick requires concentration, and that's how I do my job.”* Sophie described skating as “*therapy and art,”* noting that it “*gets my creative spark up.”* For her, learning a line or creating skate-themed art are parallel practices that affirm her identity as both an artist and a skater. These reflections underscore how skateboarding becomes a medium for enacting personhood and self-definition, particularly within a social context that often marginalizes youths' voices. Oliver linked skateboarding to Nairobi's idiom of *tarmacking*. “*When there is no kazi, we tarmack with boards,”* he laughed. “*It keeps us moving. We call it ku-tarmack na skateboard* (tarmacking with a skateboard)*, it's like you're looking for work, but you're also doing something creative.”* This reflection ties skateboarding to the everyday hustle of Nairobi's youth, who often spend hours walking the city in search of income. In this case, *tarmacking* is repurposed as a playful practice of self-discovery and joy, extending beyond mere survival.

Hope, too, is present in Nairobi's skate spaces—not as abstraction, but as something lived and enacted through repetition, rhythm, and return. It surfaces in small moments: in the push of wheels against asphalt and in the quiet persistence of showing up. Charlotte, a 19-year-old student, reflected: “*When I'm stressed from kupiga hustle, I go to the flyover and just ride; the breeze and the rhythm make me feel at home even when my pockets are empty.”* For Jessica, hope was learned through repetition: “*It's like learning how to tarmack—you keep trying, even when you get nothing in return.”* These reflections align with Clark and Sayers' (42) research on skateparks as communities of care for girls and non-binary youth, which shows that skateboarding can support well-being through shared practice and mutual support. They also echo the pedagogical insights from *Skate/Worlds*, where learning unfolds through informal and embodied practices rather than formal instruction. As Hölsgens (43) notes, a beginner “*does not learn to skate by taking lessons from a coach or instructor, but simply by doing,”* which involves observing peers, watching skate videos, and engaging in an unending process of trial-and-error. Building on this, Sayers (44) highlights that learning in skateboarding is rooted in embodied, symbolic, and social ways of knowing, which occur through participation, repetition, and shared experiences outside traditional teaching structures. Similarly, Bäckström (45) frames skateboarding as a form of *movement literacy*, in which skill acquisition emerges through feel, exploration, embodied repetition, and peer interaction. Together, these perspectives underscore how skateboarding fosters distinctive modes of learning that are experiential, collective, and self-directed (43–45). But these moments go beyond care as recovery—they express a form of embodied hope, grounded in motion, relation, and imagination. As Erica Violet Lee (11) writes, “*Hope, then, is knowing there is more to living than surviving; believing that some worlds must exist for us beyond survival. Even when we must piece those worlds together from gathered scraps, slowly building incandescent ceremonies out of nothing but our bodies, our words, and time.”* Skateboarding, in this context, becomes one such ceremony, a way of assembling *otherwise* futures from the fragments of urban neglect. Through falling and rising, through rhythmic repetition, skaters build spaces not only for survival, but for imagination, belonging, and becoming.

Situating these accounts within Nairobi's broader socio-cultural landscape, self-care for young skateboarders emerges not as an individualistic retreat, but as a relational and historical practice of becoming. Scholars of Kenyan urban youth highlight idioms like *tarmacking*—literally walking the streets to hustle for work—as central to understanding how movement and aspiration intertwine in contexts of unemployment and uncertainty (3, 4). Skateboarding retools this hustle into a form of embodied reflection: carving lines through potholed avenues becomes both a metaphor and a method for navigating precarity. In these improvisations, skaters care for their own neglected selves, reinstating their worthiness through reclaimed joy, rhythm, and presence.

Here, self-care is not about withdrawal but about sustaining dignity and possibility amid conditions that foreclose both. The skateboard becomes a tool for grounding, persistence, and self-definition—a way to create momentum when so much else is stalled. The board offers not escape but rhythm; not mastery but repetition; not spectacle but the quiet confidence of trying again. Each push and fall becomes part of a steady cadence of practice—for example, one skater was observed attempting a rail grind six times in succession, picking up speed and adjusting each time until he nearly stuck it on his seventh try. Skateboarders rarely quit when they fail; they simply rest, refocus, and try again. In this engaged flow, time itself seems to vanish as participants report losing awareness of hours passing on the board (46). As Oliver put it, *“When I’m skating, two hours pass like ten minutes. But when I’m tarmacking, even one hour feels endless.”*. In this way, skateboarding creates an alternative temporality—a rhythm of perseverance and flow that collapses anxious waiting into brief cycles of effort and renewal.

Such repurposing also underscores the global entanglements of Nairobi's skate scene: social media tutorials, international competitions, and diasporic influences circulate alongside local idioms, producing hybrid visions of possibility. In this sense, skateboarding is not simply an imported sport but a vernacular technology for assembling new futures.

### 3.2 Gendered expressions of care and feminist world-making

Despite growing visibility, female skateboarders in Nairobi continue to navigate persistent gender biases that structure both the culture of skateboarding and the urban spaces in which it unfolds. Emily recalled being told that “*this is a men's thing,”* but countered confidently that “*women are proving they can shred just as hard*.” Jessica added, “*There is, of course, some prejudice… but we are making progress,”* while Olivia explained, “*People say ‘women can get hurt… they're afraid to see you fall. These perceptions limit so many women because they're made to feel like they are less.”* Olivia continued, “*the solo girl, that's how I used to arrive, now I realized that I show up as a skateboarder, no gender.”* Similarly, Charlotte added, “*The more you challenge perceptions, the more inclusive it becomes.”* Such experiences reflect what Paechter et al. (47) describe as the performative and emotional labor required of young women in masculinized sports cultures, where simply showing up demands the constant negotiation of legitimacy. However, as these skaters push against entrenched narratives that mark risk and physicality as male, their presence also exposes how urban space is structured through colonial legacies that deny complete visibility and mobility to gendered bodies. Jessica connected skating to her *mtaa* (street) identity. “*On the streets, we say kibera ni shule (Kibera is a school)”,* she noted, “*Skating teaches you street sense and how to look out for each other. People are shocked to see girls skating, but we're from here; this is our mtaa.”* This highlights how gendered belonging is expressed through local idioms that connect place, knowledge, and communal care. As women skaters validate their presence in previously exclusionary spaces, they prescribe new social norms that invalidate patriarchal spatial codes. Thus, the practices of women skaters operate as a decolonial form of placemaking, allowing for an *otherwise* form of communal space.

Fieldnote excerpt:

“On a Sunday early afternoon, about 15 skaters gathered under the flyover. Sweat darkened their shirts as they took turns inventing trick lines on the cracked asphalt. Children from the bus stop wandered over to watch, clapping in delight when a skateboarder landed a clean kickflip. In a corner, 22-year-old Emily adjusted her helmet—one of the few girls present; she drew murmurs of respect after fearlessly attempting a drop-in from a ledge. In that moment, the derelict parking lot felt transformed into a supportive arena, its graffiti-scarred walls echoing with laughter and the rattle of wheels.”

These intimate affective encounters, of sound, gesture, shared joy, and risk, continue to echo affective economies, where emotions bind bodies together in ways that make people matter to one another. In essence, an affective collectivity is generated, in which solidarity is not only imagined but felt and enacted through shared emotional experience. These practices continue to resonate with the decolonial urbanism framework in Nairobi, where communal skate sessions become rituals that rewrite colonial geographies and dismantle the colonial divides through physical interaction. The *solo girl* thus transforms into a community member, bridging to a more general ethos of totality and spatial justice.

Women and non-binary skaters in this study emphasized the ways skateboard gatherings functioned as spaces of collective care. Emily recounted how older skaters helped her adjust her trucks and taught her how to fall safely: “*The boys here treat me like a sister, not as someone who should stay at home.”* Such experiences resonate with Clark and Sayers' (42) notion of skateparks as communities of care for marginalized genders. Similarly, the DIY peer support documented in *Skate/worlds* demonstrates how mentorship and care work circulate within skateboarding communities (48). Through everyday rituals of skating together, mentoring newcomers, and reclaiming neglected space, Nairobi's women and non-binary skaters are not just learning tricks; they are building a shared world. “*We've got all-girl skate sessions. It's been about shining a light on female talent,”* Ben explained, while Emily described these gatherings as helping her “*find courage”* and “*to go out and skate in the streets.”* These sessions serve as safe havens, where supportive peer environments allow marginalized skaters to feel seen, acquire skills, and challenge limiting gender norms (42). Rather than merely protective, they are productive; they are brave spaces where vulnerability becomes a site of power and growth. Initiatives like She Skates Kenya or GirlSkate Nairobi frame skateboarding through a feminist ethics of kinship, centering marginalized voices and rewriting who belongs in the city. Vivoni and Folsom-Fraster (32) demonstrate that street skateboarding forms communities grounded in mutual support and inclusive forums. Through collective spatial appropriation and redefinition, skaters remake exclusionary public spaces. These care-rooted practices advance spatial justice. Additionally, Paechter et al. (47) highlight how gender-inclusive programs stake a right to the city through embodied presence. These efforts respond directly to what Wilkes and Hird (49) describe as colonial residues, material and symbolic barriers that still shape post-colonial urban space. In Nairobi, these barriers are not simply sidestepped but challenged, as women and queer skaters refuse to enter quietly and instead build something new in the cracks. This emotional ecology, grounded in cheering, spotting, and creating space for one another, enacts Lorde's (41) vision of radical self-care. These small rituals of support and affirmation reconfigure the space itself, where offering tips or building a ramp becomes a form of feminist urban practice. As D'Orazio (50) shows, such spatial tactics of curating sessions or sharing gear are not only adaptive but refusals to be erased or excluded. What emerges is not just inclusion, but the slow transformation of skateboarding into a feminist, decolonial terrain of care, creativity, and urban belonging through the otherwise.

In a city where patriarchal norms circumscribe women's mobility, female skaters described skate sessions as acts of defiance and mutual protection. Olivia explained that when men yelled at her to go home, her crew stood around her and continued to skate, demonstrating that “*this space is ours.”* Such practices illustrate how gendered expressions of care in Nairobi are deeply relational and political, challenging prevailing norms while forging new solidarities. To fully appreciate the gendered contours of care in Nairobi's skate scene, these narratives must be situated within East Africa's colonial and post-colonial gender regimes. Scholars note that colonial law and mission education instituted rigid gender roles that continue to circumscribe women's mobility and bodily autonomy (6). By occupying streets and public plazas with boards under their feet, female and non-binary skaters contest these inherited divisions, refusing the ideological domestication of their bodies. Their experiences also reveal intersections with class: whereas middle-class women may face moral surveillance, those from low-income settlements encounter material barriers such as lack of equipment, safe transit, and familial support. Their solidarity across these divides signals what Willing and Pappalardo (51) call an *ethic of reciprocity*—a commitment to mutual uplift rooted in shared vulnerability. Yet care here is not sentimental; rather, it is often forged through friction and negotiation. In our interviews, several skaters described a recent controversy within a women-led collective, where community funds meant for skate clinics were allegedly misused. “*We trusted the organizers, but the money for our workshops disappeared,*” one participant explained. The ensuing conflict involved heated meetings, demands for accountability, and a complete restructuring of leadership. Another skater reflected that care, in this context, meant “*calling each other out and making sure no one is benefiting at others' expense.”* These tensions underscore that care is often forged through friction and negotiation; they support Willing and Pappalardo's (51) argument that global empowerment narratives can obscure the ways in which local patriarchy and structural violence shape community projects. By confronting power imbalances within their own ranks, Nairobi's skaters turn care into a political practice grounded in mutual accountability rather than sentiment alone.

### 3.3 Community care, commons, and ecological care

Community care in Nairobi's skate scene extends beyond interpersonal support to include collective environmental stewardship and the construction of urban commons. Skaters from diverse neighborhoods join not only to share tricks but to clean, rebuild, and re-imagine derelict spaces. The material labor of caring for place emerges in vernacular practices of environmental stewardship. During a clean-up along the Nairobi River, Ben reflected, “*We call it kupiga safi mtaa (cleaning the neighborhood). It's not just about skateboarding; we make our home livable.”* Emily added, “*We used to pass by garbage and think hakuna mtu anajali (no one cares). Now we pick it up and skate over it*.” These acts align with what O'Connor and colleagues describe as skaters becoming caretakers of grey spaces: repurposing polluted corridors into playable terrains while being aware that skateboarding itself produces environmental impacts. Local idioms, such as *“kujenga jukwaa la mtaa”* (building a stage for the hood), articulate a relational ethic of care rooted in Nairobi's street cultures. Instead of viewing wastelands as external to urban life, skaters treat them as the very matter of community. They animate neglected infrastructures with creativity and intent, transforming grey, post-colonial spaces into vibrant terrains of meaning. Ramp-building from scavenged materials, graffiti art, and inclusive skate sessions for often-excluded groups are not only aesthetic interventions but also political acts of spatial re-imagining. By doing this, they show care for spaces that others have written off—abandoned lots, crumbling surfaces, and polluted city edges—treating them not as voids, but as places still capable of holding life, relation, and possibility. Their movements and gestures reclaim these wastelands as sites of attachment, attention, and slow transformation. As Lee writes, “*we understand that there is nothing and no one beyond healing. So we return again and again to the discards, gathering scraps for our bundles, and we tend to the devastation with destabilizing gentleness, carefulness, softness.”* In this spirit, skateboarding becomes not only a mode of mobility or expression, but a quiet, persistent way of tending to the urban *otherwise*.

However, critical scholarship on grey spaces highlights their environmental and political ambiguities. Hölsgens and Glenney (27) similarly contend that without grey spaces there is no skate culture: the practice is “*decidedly modern and complicit to ecological damage”,* yet new eco-conscious movements seek to make skateboarding more caring and sustainable. O'Connor et al. (10) argue that skateboarding is a *polluted leisure* whereby skaters are complicit in neoliberal infrastructural waste yet cultivate alternative practices of sustainability. Glenney and Dubois (19) similarly describe skateparks as contested sites where pollution, colonial legacies, and environmental justice coalesce. Nairobi's skaters embody these tensions. They build DIY obstacles from scavenged wood and concrete, yet these materials rely on extractive industries; they champion sustainability but must navigate a city whose green spaces are scarce. In this sense, community care is not purely restorative but negotiated within the contradictions of urban modernity. Acts of repairing rails or sweeping dust off a plaza are simultaneously practices of dwelling and survival, as well as environmental consciousness and complicity.

In Mexico, Buchetti (52) documents how skaters transform waste into communal skateparks, blending environmental activism with Indigenous rituals of care and memorial. Glover et al. (53) observe that at Toronto's Bentway, skateboarders engage in *gentle activism*, animating public spaces through arts and community events without directly confronting authorities. These studies underscore that environmental care in skateboarding is always socially mediated. In Nairobi, similar gentle activism is evident in volunteer-driven improvements to Shangilia Skatepark and the collective beautification of riverbanks. William recounted how “*turning a parking lot into ramps”* attracted vendors, parents, and passersby, creating a lively commons. Such transformations are part of a broader urban commons movement, identified by Zapata Campos et al. (54) as everyday collective resistance. Nairobi skaters thus practice an improvised environmentalism; they mix play with clean-up drives, art with waste removal, and mutual aid with infrastructure building, producing a commons that is at once aesthetic, social, and ecological.

Nevertheless, these community efforts encounter resistance from private security and law enforcement. Participants described being harassed or chased from plazas and rooftops under the pretext of protecting property. “*The guards around the malls or wherever… they should stop chasing us around. We're not doing anything wrong—we are just skateboarding,”* Emily said. Ben reflected that these confrontations feel less about safety than about marking control: “*It's like they want to show us we don't belong. But skating there is our way of showing we do.”* Several recounted being pushed out of public spaces, threatened with arrest, or accused of loitering—despite no laws being broken and no damage done. These cat-and-mouse dynamics reveal how authority figures manage space not as a shared commons but as controlled territory, illustrating what Dickinson et al. (31) describe as the *aesthetic order* of public spaces, where skateboarding is policed as disorderly, even when it produces no harm. The skateboarders, many of whom come from Nairobi's lower-income neighborhoods, understand these confrontations as daily injustices. “*We keep skating, and they chase us,”* Emily explained, “*well, we are claiming this spot, and we keep skating here.”* By persisting, they assert their right to occupy and enliven spaces that were not designed for them, challenging both formal regulations and informal norms that exclude youth from the city's privatized landscapes. These confrontations illustrate how, as Dickinson et al. (31) note, public spaces are ordered to exclude certain bodies and activities. Skateboarders respond not with violent protest but through persistence: returning to the same spots, sweeping away debris, repainting surfaces, and reusing discarded materials. These acts, which are both political and prefigurative, illustrate what Glover and colleagues (53) refer to as gentle activism. They insist on belonging in a city that often views them as out-of-place.

The intersection of community care and ecological stewardship emerges as a distinguishing feature of Nairobi's skate culture. In contrast to skate communities in the global North, where environmental activism often centers on climate advocacy or green consumption, Nairobi's skaters practice what might be called decolonial environmentalism: their care for waste landscapes is inseparable from struggles over land, life, and sovereignty. Here, decolonial environmentalism is material and relational as it emerges from the necessity of making spaces livable under conditions of infrastructural neglect. The skaters engage with pollution not as an abstract issue but as a felt reality requiring collective action. Histories of colonial extraction has left behind toxic dumpsites, channeled sewage into informal settlements, and relegated public amenities to decay; while contemporary neoliberal urbanism continues to exclude youth from sanitized commercial spaces (3). By cleaning riverbanks, repurposing scrap wood into ramps, and repainting walls with East African motifs, skateboarders refuse the designation of these places as *wastelands*. Their actions echo Indigenous-led movements across the globe that reclaim *grey spaces* as sites of cultural memory and resilience (19, 55). Yet Nairobi's scene is distinct in its mobilization of Sheng and Kiswahili idioms—*kupiga safi mtaa* (cleaning the neighborhood), *kujenga jukwaa la mtaa* (building a stage for the hood)—by naming and thus giving meaning to the labor. Such linguistic innovations signal a shift from viewing environmental work as charity to understanding it as a collective responsibility and a matter of spatial justice.

## 4 Conclusion

This study offers a situated, relational account of skateboarding in Nairobi, grounded in the lived experiences of a core group of active participants. In line with decolonial feminist methodologies, our aim was to understand how everyday practices of movement, care, and community take shape under conditions of structural neglect. The findings extend feminist and decolonial urban theory by showing how Nairobi's skateboarders engage in everyday practices of world-making grounded in care, refusal, and collective presence. Skateboarding emerges as an embodied and affective pedagogy where emotional expression, self-affirmation, and mutual recognition are cultivated through repetition and shared joy. Drawing on decolonial feminist insights, the youth enact survival not as individual persistence, but as relational, spatial practice shaped by care and solidarity. Affective collectivities—through cheering, mentoring, and skating together—build relational infrastructures that disrupt fragmentation and reframe marginalized space as grounds for belonging. Through these shared acts, skaters enact an *otherwise* form of being together in difference that exceeds logics of exclusion and disposability. As they *care for wastelands*—both derelict land and each other—they reframe abandonment not as decay, but as an invitation for creativity and communal stewardship. Gendered experiences deepen these practices, as women and non-binary skaters assert presence through supportive networks and radical self-care, in defiance of colonial and patriarchal spatial orders. Ultimately, Nairobi's skateboarders re-imagine space, self, and community not through possession or power, but through movement, affect, and care for what has been cast aside. The cohort's diversity itself illuminated Nairobi's urban precarity. Female skaters described harassment and safety fears—a gendered precarity of Nairobi's streets—while male peers expressed concern over unemployment and limited recreational space. These underscore urban precarity as multifaceted, blending economic uncertainty with social vulnerability. The convergence of skaters from Kibera's informal settlements and Nairobi's middle-class estates temporarily dissolves class barriers, revealing how each group navigates instability differently. Those from low-income areas improvise communal support to offset material insecurity, while female skaters challenge patriarchal norms to claim space. Participant diversity thus reveals a layered urban condition—a tapestry of insecurity and creativity shaped by gender, class, and ethnicity.

At the same time, the study's limitations highlight key directions for future research. Participants were primarily drawn from skaters already embedded in collectives like She Skates Kenya, GirlSkate Nairobi, and the Skateboarding Society of Kenya. While this enabled rich, reflective narratives, it may have underrepresented more precarious or peripheral experiences—such as those of dropouts, younger skaters, or individuals without access to such networks. These absences underscore how visibility is shaped by access and relational ties, affirming the ongoing ethical imperative to listen from the margins in decolonial research. Additionally, most interviews were conducted in English, which is not the first or most affective language for many participants. Nuances embedded in Sheng, Kiswahili, or other mother tongues may have been softened or lost, limiting access to the full emotional texture of participants' world-making. This reflects broader colonial residues in research infrastructures. Future work would benefit from multilingual approaches that center linguistic intimacy as a form of epistemic justice.

What emerges, then, is not simply a subculture, but a living archive of how to move with joy, creativity, and care against—and within—the persistent conditions of disposability. Nairobi's skaters transform broken infrastructures into sites of solidarity and imagination, while living within layered violences of coloniality and neglect. Agency begins with a skateboard—an object that becomes a tool for subjectivity, collective authorship, and the quiet remaking of the city.

## Data Availability

The datasets presented in this study can be found in online repositories. The names of the repository/repositories and accession number(s) can be found in the article/Supplementary Material.

## References

[1] Karijo E Wamugi S Lemanyishoe S Njuki J Boit F Kibui V Knowledge, attitudes, practices, and the effects of COVID-19 among the youth in Kenya. BMC Public Health. (2021) 21(1):1–13. 10.1186/s12889-021-11067-2 34053442 PMC8164891

[2] giz. Youth Employment in the Agri-Food Sector in Western Kenya. Available online at: https://www.giz.de/en (Accessed August 19, 2025).

[3] Rahier N. ‘Tarmacking is Tedious’: Navigating City Pressures in Nakuru, Kenya.’ City (April 23, 2024). Available online at: https://www.tandfonline.com/doi/abs/10.1080/13604813.2024.2342092 (Accessed August 19, 2025).

[4] Thieme TA. The “hustle” amongst youth entrepreneurs in Mathare’s informal waste economy. J East Afr Stud. (2013) 7(3):389–412. 10.1080/17531055.2013.770678

[5] Chułek M. Hustling the mtaa way: the brain work of the garbage business in Nairobi’s slums. Afr Stud Rev. (2020) 63(2):331–52. 10.1017/asr.2019.46

[6] Van Stapele N. Providing to belong: masculinities, hustling and economic uncertainty in Nairobi ‘ghettos’. Africa (Lond). (2021) 91(1):57–76. 10.1017/S0001972020000844

[7] Martin AM Bezemer PM. The concept and planning of public native housing estates in Nairobi/Kenya, 1918–1948. Plan Perspect. (2019) 35:609–34. 10.1080/02665433.2019.1602785

[8] Schramm S Bize A. Planning by exception: the regulation of Nairobi’s margins. Plan Theory. (2023) 22(3):316–37. 10.1177/14730952221137706

[9] Harrison P Croese S. The persistence and rise of master planning in urban Africa: transnational circuits and local ambitions. Plan Perspect. (2023) 38(1):25–47. 10.1080/02665433.2022.2053880

[10] O’Connor P Evers C Glenney B Willing I. Skateboarding in the anthropocene: grey spaces of polluted leisure. Leis Stud. (2023) 42(6):897–907. 10.1080/02614367.2022.2153906

[11] Lee EV. In Defence of the Wastelands: A Survival Guide—GUTS (2016). Available online at: https://gutsmagazine.ca/wastelands/ (Accessed May 9, 2025).

[12] Zittel E. Nairobi on Board: Skateboarders’ Sensory Experiences of Community, Body, and Space (2023). Available online at: http://lup.lub.lu.se/student-papers/record/9135164 (Accessed August 31, 2024).

[13] Esene S. Nine African Skating Communities Championing Women and Girls | OkayAfrica (2024). Available online at: https://www.okayafrica.com/women-skating-communities-africa/ (Accessed May 20, 2025).

[14] Hölsgens S Glenney B. Skateboarding and the Senses: Skills, Surfaces, and Spaces. 1st ed. London: Routledge (2024). 10.4324/9781003510642

[15] Romero N Miles D. Anticolonial skate pedagogy: skateboarding as decolonising education. In: Hölsgens S Ong A, editors. Skate/worlds: New Pedagogies for Skateboarding. Groningen: University of Groningen Press (2025). Chapter 10. p. 217. Available online at: https://books.ugp.rug.nl/ugp/catalog/book/208 (Accessed May 20, 2025).

[16] Geckle B. Symbolic sovereignty and the decolonizing and indigenizing potentials of skateboarding. Emerg Media. (2025) 3(1):23–37. 10.1177/27523543241310860

[17] Kamper D Williams N. Skateboarders of color and the (co-)emergence of the DIY ethos in skateboarding. Sport Soc. (2025) 28(2):316–31. 10.1080/17430437.2025.2457825

[18] Hölsgens S Ong A. Skate/worlds: New Pedagogies for Skateboarding. Groningen: University of Groningen Press. University of Groningen Press (2025).

[19] Glenney B Dubois P. Skateparks, pollution, and decolonisation. Leis Stud. (2025) 44:923–37. 10.1080/02614367.2025.2462101

[20] Mignolo WD. Coloniality and globalization: a decolonial take. Globalizations. (2020) 18:720–37. 10.1080/14747731.2020.1842094

[21] Walsh CE. Decolonial pedagogies walking and asking. Notes to Paulo Freire from AbyaYala. International J Lifelong Educ. (2015) 34(1):9–21. 10.1080/02601370.2014.991522

[22] Maldonado-Torres N. On the coloniality of being: contributions to the development of a concept. Cultural Studies. (2007) 21(2–3):240–70. 10.1080/09502380601162548

[23] Mignolo WD Walsh CE. On Decoloniality. On Decoloniality (July 25, 2018).

[24] Mbembe A. Out of the Dark Night: Essays on Decolonization. New York: Columbia University Press (2021). 10.7312/mbem16028

[25] hooks bell. All about love: new visions. World Lit Today. (2018) 60(3):487. Available online at: https://openurl.ebsco.com/contentitem/cat05074a:malmo.b3035699?sid=ebsco:plink:crawler&id=ebsco:cat05074a:malmo.b3035699

[26] Tsipis A. Urban Rides, Social Tides : Skateboarding’s Influence on Youth Empowerment and Urban Cultural Dynamics in Nairobi, Kenya. Malmö: Malmö University (2024). Available online at: https://urn.kb.se/resolve?urn=urn:nbn:se:mau:diva-71303 (Accessed May 20, 2025).

[27] Hölsgens S Glenney B. Skateboarding and the Senses: Skills, Surfaces, and Spaces. 1st ed. London: Routledge. 10.4324/9781003510642

[28] Sverdlik A Gichuiya LN Glück Z Kanyinga K Kimari W Magero J Nairobi: City Report 2025. Available online at: http://www.african-cities.org (Accessed May 20, 2025).

[29] Bagayoko M Akeyo D Kadengye DT Iddi S. Understanding wealth transitions among households in urban slums of Nairobi: a multi-state transition modelling approach. Glob Epidemiol. (2020) 2:100037. 10.1016/j.gloepi.2020.100037 PMC10445989 37635722

[30] Bäckström Å Sand AL. Imagining and making material encounters: skateboarding, emplacement, and spatial desire. J Sport Soc Issues. (2019) 43(2):122–42. 10.1177/0193723519830463

[31] Dickinson S Millie A Peters E. Street skateboarding and the aesthetic order of public spaces. Br J Criminol. (2022) 62(6):1454–69. 10.1093/bjc/azab109

[32] Vivoni F Folsom-Fraster J. Crafting cities for all: qualitative inquiry of the street and the spatial practice of skateboarding. Cult Stud. (2021) 21(4):311–8. 10.1177/15327086211004879

[33] Skate-Aid. Support the Kids in Nairobi, Kenia | Africa | Skate-Aid e.V. Available online at: https://www.skate-aid.org/en/projects/africa/kenya-nairobi/ (Accessed August 30, 2024).

[34] Concrete Disciples. Shangilia Skatepark Nairobi Kenya—Directory and Listings—Concrete Disciples Skatepark Locator and Skate Shop. Available online at: https://www.concretedisciples.com/global-skatepark-directory/kenya/skatepark-kenia-africa/?srsltid=AfmBOorXWhyI1iJZpDhTt51aUkxyzY8ALMI26WyHJVG9X5uNA_ pchSxe&utm (Accessed May 20, 2025).

[35] UN-Habitat. SDG Indicators (2023). Available online at: https://unstats.un.org/sdgs/report/2023/ (Accessed May 9, 2025).

[36] World Bank Group. Kenya Overview: Development News, Research, Data | World Bank (2025). Available online at: https://www.worldbank.org/en/country/kenya/overview (Accessed May 20, 2025).

[37] Awoche Foundation. Awoche Foundation (2023). Available online at: https://awochefoundation.org/ (Accessed May 20, 2025).

[38] Braun V Clarke V Weate P. 5 Using Thematic Analysis in Sport and Exercise Research (2016).

[39] Glenney B Bjorke I Buchetti A. Skateboarding and the surplus value of city play. Front Sports Act Living. (2024) 6:1454274. 10.3389/fspor.2024.1454274 39553376 PMC11563835

[40] Mashreghi S. Decolonial stories of forced migrants in physical activity and sport: “We the Afghan kids.” In: De Martini Ugolotti N Caudwell J, editors. Leisure and Forced Migration: Lives Lived in Asylum Systems. London: Routledge (2021). p. 157–75. 10.4324/9780429341045

[41] Lorde A. A Burst of Light: Essays. Ithaca (NY): Firebrand Books (1988).

[42] Clark S Sayers E. Skateparks as Communities of Care: The Role of Skateboarding in Girls’ and Non-Binary Youth’s Mental Health Recovery during Lockdown (2023). Available online at: https://www.tandfonline.com/action/journalInformation?journalCode=rpcs20 (Accessed October 18, 2023).

[43] Hölsgens SRJJ. “We Belong Here” Lessons from Skateboarding. Groningen: University of Groningen press (2025). p. 7–28. Available online at: https://hdl.handle.net/1887/4254945 (Accessed August 20, 2025).

[44] Sayers E. Skate/Worlds: New Pedagogies for Skateboarding. Groningen: University of Groningen Press (2025). p. 73–94.

[45] Bäcstrom Å. Skate/Worlds: New Pedagogies for Skateboarding. Groningen: University of Groningen Press (2025). p. 145–72.

[46] Seifert T Hedderson C. Intrinsic motivation and flow in skateboarding: an ethnographic study. J Happiness Stud. (2010) 11(3):277–92. 10.1007/s10902-009-9140-y

[47] Paechter C Stoodley L Keenan M Lawton C. What’s it like to be a girl skateboarder? Identity, participation and exclusion for young women in skateboarding spaces and communities. Womens Stud Int Forum. (2023) 96:102675. 10.1016/j.wsif.2023.102675

[48] Gil A Forsyth J. Skate/Worlds: New Pedagogies for Skateboarding. in. University of Groningen Press. University of Groningen Press (2025). p. 95–120.

[49] Wilkes J Hird MJ. Colonial Ideologies of Waste: Implications for Land and Life—EuropeNow (2019). Available online at: https://www.europenowjournal.org/2019/05/06/colonial-ideologies-of-waste-implications-for-land-and-life/ (Accessed May 9, 2025).

[50] D’Orazio D. Skateboarding’s olympic moment: the gendered contours of sportification. J Sport Soc Issues. (2020) 45(5):395–425. 10.1177/0193723520928595

[51] Willing I Pappalardo A. Skateboarding Power and Change. Singapore: Springer Nature Singapore Pre Ltd. (2023). p. 1–295.

[52] Buchetti A. The grey forms of knowledge. Border scraps, secrecy and the reproduction of skateboarding in Tijuana (Mexico). Leis Stud. (2025) 44:875–91. 10.1080/02614367.2025.2455579

[53] Glover TD Munro S Men I Loates W Altman I. Skateboarding, gentle activism, and the animation of public space: CITE—a celebration of skateboard arts and culture at the bentway. Leis Activism Animation Urban Environ. (2022) 40:42–56. 10.4324/9781003328704-4

[54] Zapata Campos MJ Zapata P Pérez Reynosa J. (Re)gaining the urban commons: everyday, collective, and identity resistance. Urban Geogr. (2023) 44(7):1259–84. 10.1080/02723638.2022.2090117

[55] Buchetti A O’Connor P. Chicano park’s skateboard memorial murals: extending the sacred in polluted leisure. Leis Sci. (2025) 47(3):475–98. 10.1080/01490400.2024.2325484

